# PerR Confers Phagocytic Killing Resistance and Allows Pharyngeal Colonization by Group A *Streptococcus*

**DOI:** 10.1371/journal.ppat.1000145

**Published:** 2008-09-05

**Authors:** Ioannis Gryllos, Renata Grifantini, Annalisa Colaprico, Max E. Cary, Anders Hakansson, David W. Carey, Maria Suarez-Chavez, Leslie A. Kalish, Paul D. Mitchell, Gary L. White, Michael R. Wessels

**Affiliations:** 1 Division of Infectious Diseases, Children's Hospital Boston, Massachusetts, United States of America; 2 Harvard Medical School, Boston, Massachusetts, United States of America; 3 Novartis Vaccines and Diagnostics, Siena, Italy; 4 Department of Pathology and Comparative Medicine, University of Oklahoma Health Sciences Center, Oklahoma City, Oklahoma, United States of America; 5 Clinical Research Program, Children's Hospital Boston, Boston, Massachusetts, United States of America; Schepens Eye Research Institute, United States of America

## Abstract

The peroxide response transcriptional regulator, PerR, is thought to contribute to virulence of group A *Streptococcus* (GAS); however, the specific mechanism through which it enhances adaptation for survival in the human host remains unknown. Here, we identify a critical role of PerR-regulated gene expression in GAS phagocytosis resistance and in virulence during pharyngeal infection. Deletion of *perR* in M-type 3 strain 003Sm was associated with reduced resistance to phagocytic killing in human blood and by murine macrophages *in vitro*. The increased phagocytic killing of the *perR* mutant was abrogated in the presence of the general oxidative burst inhibitor diphenyleneiodonium chloride (DPI), a result that suggests PerR-dependent gene expression counteracts the phagocyte oxidative burst. Moreover, an isogenic *perR* mutant was severely attenuated in a baboon model of GAS pharyngitis. In competitive infection experiments, the *perR* mutant was cleared from two animals at 24 h and from four of five animals by day 14, in sharp contrast to wild-type bacteria that persisted in the same five animals for 28 to 42 d. GAS genomic microarrays were used to compare wild-type and *perR* mutant transcriptomes in order to characterize the PerR regulon of GAS. These studies identified 42 PerR-dependent loci, the majority of which had not been previously recognized. Surprisingly, a large proportion of these loci are involved in sugar utilization and transport, in addition to oxidative stress adaptive responses and virulence. This finding suggests a novel role for PerR in mediating sugar uptake and utilization that, together with phagocytic killing resistance, may contribute to GAS fitness in the infected host. We conclude that PerR controls expression of a diverse regulon that enhances GAS resistance to phagocytic killing and allows adaptation for survival in the pharynx.

## Author Summary

Group A *Streptococcus* (GAS) is the leading cause of bacterial pharyngitis (strep throat) in children. It is also associated with the life-threatening conditions of necrotizing fasciitis, streptococcal toxic shock, and the post-streptococcal autoimmune syndrome of rheumatic fever and heart disease. The peroxide response regulator PerR is thought to enhance GAS pathogenesis by coordinating adaptive responses of the bacteria in oxidative environments, but its specific contribution to GAS adaptation and virulence at various host sites during infection is poorly understood. In studies comparing phagocytic killing resistance of wild-type and a *perR* mutant strain of GAS, we find that PerR contributes to bacterial survival in the oxidative environment of phagocytes either during growth in human blood or during interaction with mouse macrophages. Moreover, our studies in a primate model of human pharyngitis suggest that PerR is critical for pharyngeal infection, as PerR mutant bacteria were able to establish infection in one of five inoculated animals in contrast to wild-type bacteria, which established infection in all five animals used. Initial investigation into the genes controlled by PerR in GAS identified a range of bacterial products that may contribute to GAS survival and virulence during infection.

## Introduction

Group A *Streptococcus* (*Streptococcus pyogenes* or GAS) is a strictly human pathogen that causes a spectrum of disease ranging from superficial infection of the pharyngeal mucosa and the skin to invasive infection of deep tissues and the bloodstream. Like other lactic acid bacteria, GAS lacks oxidative phosphorylation machinery and instead obtains energy by fermentation. Despite this lack of oxygen requirement, GAS has evolved to grow rapidly in the oxygen-rich human host environment and to resist killing by reactive oxygen species (ROS), such as hydrogen peroxide (H_2_O_2_) and superoxide (O_2_^−^), which are formed by reduction of atmospheric oxygen or produced by phagocytes upon activation of the host inflammatory response. Unlike some other Gram-positive pathogenic bacteria, GAS lacks catalase (a heme-containing peroxidase) that degrades H_2_O_2_ to H_2_O and O_2_, but produces several alternative peroxidases that degrade organic and inorganic peroxides formed in the presence of O_2_ or as a consequence of inflammation. Among these, the NADH peroxidase (*npr*), alkyl hydroperoxidase (*ahpC*) and glutathione peroxidase (*gpoA*) have been shown to contribute to GAS aerotolerance and ROS detoxification during culture *in vitro* as well as during infection in mice [1]–[4]. Resistance to O_2_^−^ toxicity is mediated by superoxide dismutase (*sodA*), which converts O_2_^−^ to H_2_O_2_, which is detoxified in turn by peroxidases. Superoxide dismutase is also critical for GAS aerotolerance and survival in oxidative environments, as a *sodA* mutant was not only more sensitive to O_2_^−^-generating agents but also was unable to grow under standard aerobic growth conditions [5].

GAS adaptive responses to oxidative stress are coordinated, at least in part, by the peroxide stress response regulator PerR, which belongs to the Fur (ferric uptake repressor) super-family of metal-binding transcriptional regulators [2],[6]. PerR was originally shown to control oxidative stress responses in *Bacillus subtilis* through direct binding of conserved promoter sequences known as Per boxes [7]. In the absence of oxidative conditions, PerR represses PerR regulon expression in *B. subtilis* by remaining bound to Per boxes in PerR-regulated promoters. In this form, PerR contains a structural zinc atom, as well as a regulatory ferrous ion, binding of which is coordinated by several amino acids including three histidines. Under oxidative conditions, the bound ferrous ion catalyses oxidation of two of three histidine residues, promoting release of oxidized PerR from target promoters and de-repression of the PerR regulon [8],[9].

In *B. subtilis* and *Staphylococcus aureus*, PerR controls expression of peroxidases (catalase, alkylhydroperoxide reductase) among other genes, and coordinates oxidative stress responses and iron homeostasis [7],[10],[11]. Such coordinate control is critical for bacterial survival as free intracellular iron reacts with H_2_O_2_ to form highly oxidizing hydroxyl radicals (HO^.^) in what is known as the Fenton reaction. Certain studies have suggested that expression of known peroxidases and all three described iron acquisition systems of GAS [12]–[14] is PerR-independent, and therefore, PerR regulation in GAS differs from other Gram-positive species [2],[4],[15]. However, decreased expression of superoxide dismutase and the MtsABC iron transport system has been reported in an M-type 1 GAS *perR* mutant [6]. More recent evidence suggests that PerR may be involved in coordinating oxidative stress responses and metal homeostasis in GAS. Characterization of the PerR regulon in M-type 5 GAS, by comparing a *perR* mutant transcriptome to that of wild-type during mid-exponential phase growth, suggested strong regulation (3-fold or higher) of six genes by PerR; however, only *pmtA*, a gene encoding a putative metal efflux protein, was proposed to be under direct PerR control [16]. Interestingly, the previously reported enhanced resistance of *perR* mutants to H_2_O_2_ killing *in vitro* was attributed to *pmtA* overexpression, as mutation of *pmtA* in the *perR* mutant background led to lower H_2_O_2_ resistance, and single *pmtA* mutants were over 10-fold more sensitive to H_2_O_2_ killing than wild-type GAS. Deregulation of the remaining five genes in the M-type 5 *perR* mutant was proposed to be an indirect effect of metal starvation resulting from *pmtA* upregulation, and was thought to be mediated by a second transcriptional regulator protein, AdcR, although regulation of *adcR* itself by PerR was not shown [16]. Even though PmtA was associated with resistance to H_2_O_2_ killing *in vitro*, no PerR-regulated gene identified in that strain, including *pmtA*, was linked to GAS survival and virulence *in vivo*.

Despite their increased resistance to H_2_O_2_ challenge, M-type 1 and M-type 5 *perR* mutants of GAS show sensitivity to superoxide *in vitro* and significant loss of virulence following subcutaneous or intraperitoneal inoculation in mice [2],[4],[6]. However, the mechanism through which PerR contributes to GAS virulence and the specific contribution of PerR-regulated gene expression to bacterial survival at particular host sites during infection remains unknown. In this study, we demonstrate a critical role of PerR-regulated gene expression in GAS resistance to phagocytic killing and in pharyngeal colonization in primates. In addition, using genomic microarrays, we find that the GAS PerR regulon is substantially more extensive and more diverse than previously appreciated, a finding that might explain the pivotal role of PerR in GAS phagocytosis resistance and pharyngeal infection.

## Results

### Construction of a *perR* mutant in M-type 3 GAS strain 003Sm

**Figure 1:**
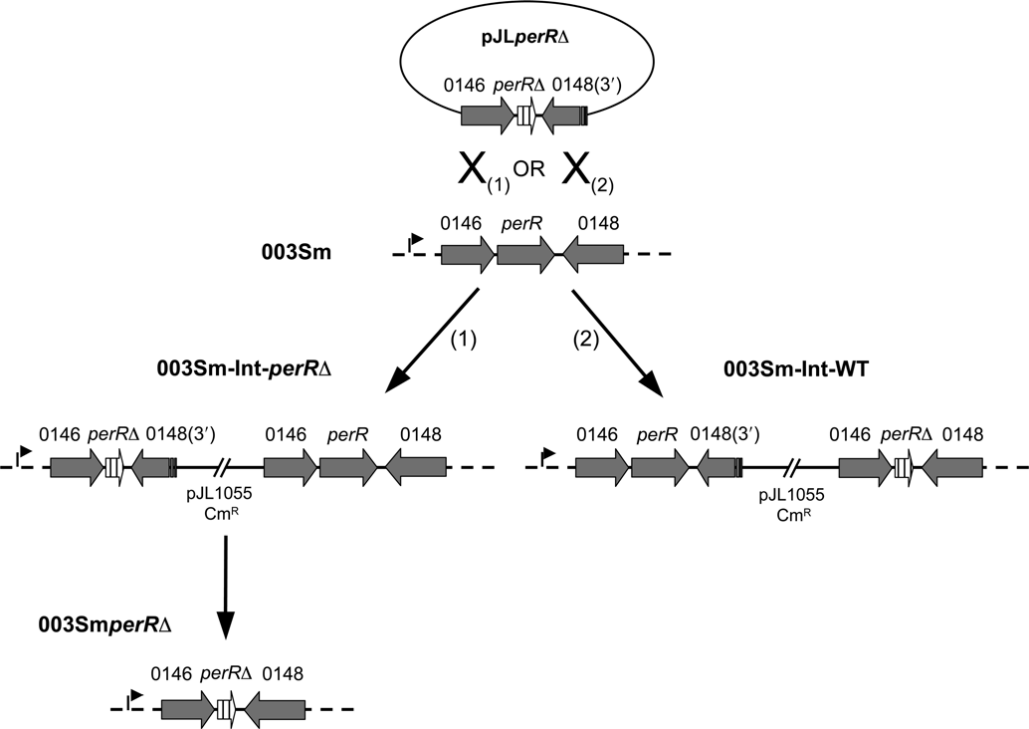
Mutagenesis of *perR* (*spyM3_0147*) in M-type 3 GAS strain 003Sm. Chromosomal integration of the temperature-sensitive plasmid pJL*perR*Δ upstream or downstream of *perR* resulted in integrant strains 003Sm-Int-*perR*Δ and 003Sm-Int-WT that exhibited *perR* mutant and wild-type phenotype, respectively. In addition to the truncated *perR* copy, the deletion construct on pJL*perR*Δ encompassed the complete coding sequence of *spyM3_0146* located upstream of *perR* and the 3′-end sequence of *spyM3_0148* located downstream of *perR* in opposite orientation. The recombination event (X_1_) in *perR* mutant strain 003Sm-Int-*perR*Δ separated the wild-type *perR* copy, still present on the chromosome, from its native promoter (depicted with an arrow upstream of *spyM3_0146*) resulting in loss of PerR expression. In strain 003Sm-Int-WT, recombination in the 3′-end of *spyM3_0148* (X_2_) did not separate *perR* from its natural promoter, thereby allowing PerR expression and wild-type phenotype. To derive the deletion mutant strain 003Sm*perR*Δ, excision of the plasmid from the chromosome with simultaneous recombination downstream of *perR* was achieved during growth of integrant strain 003Sm-Int-*perR*Δ at 30°C.

To study the role of *perR* in GAS virulence and pathogenesis, a *perR* deletion mutant was derived from strain 003Sm, a spontaneous streptomycin resistant (Sm^R^) variant of wild-type M-type 3 strain DLS003 [17]. Mutagenesis was designed based on the genome sequence of M-type 3 strain MGAS315 [18]. An in-frame 426 bp deletion of the 468 bp coding sequence of *perR* (*spyM3_0147*) was constructed in the temperature-sensitive shuttle vector pJL1055, and the resulting plasmid pJL*perR*Δ was used to generate the *perR* deletion mutant strain 003Sm*perR*D by allelic exchange (Figure 1). The mutant exhibited wild-type growth rates under aerobic conditions during culture in THY broth or on THY-blood agar. As reported for *perR* mutants constructed in M-type 1 and M-type 5 GAS [2],[6], resistance of 003Sm*perR*DΔ to H_2_O_2_ challenge (10 mM) in liquid culture was approximately 15-fold higher than that of parent strain 003Sm, with survival rates of 23% versus 1.6%, respectively (Figure S1).

### The *perR* mutant has impaired resistance to phagocytic killing by human blood leukocytes

The involvement of PerR in oxidative stress adaptive responses of GAS *in vivo* was evaluated by testing phagocytic killing resistance of the *perR* mutant strain 003Sm*perR*Δ in the Lancefield bactericidal test [19]. Wild-type strain 003Sm and *perR* mutant 003Sm*perR*Δ were rotated end-over-end in heparinized human blood at 37°C, and survival was assessed over time. As indicated by the viable counts recovered 3 h post-inoculation, wild-type bacteria showed a net growth of 4-fold relative to the inoculum in sharp contrast to the *perR* mutant that did not exhibit any net growth over the same time period (Table 1). In control assays in which bacteria were incubated for 3 h without rotation (required for physical contact between leukocytes and bacteria for efficient phagocytosis), both mutant and wild-type GAS increased by 30 to 50-fold. Thus, the reduced proliferation of the mutant relative to wild-type bacteria in rotating blood was due to its increased sensitivity to killing by phagocytes. To further validate this result, we assessed phagocytic killing resistance of the *perR* mutant strain expressing PerR constitutively from plasmid pORI-*perR in trans*, or the *perR* mutant strain carrying empty vector pORI23 as control. For reasons that are unclear, culture of GAS on erythromycin (for maintenance of pORI-*perR* and pORI23) increased baseline survival in human blood more than 10-fold. Even so, survival of strain 003Sm*perR*Δ (pORI-*perR*) was significantly higher (1.8-fold) than that of control strain 003Sm*perR*Δ (pORI23), a finding that also supported the role of PerR in GAS phagocytic killing resistance (*P*<0.01 for comparison of survival at 3 h of *perR* mutant carrying pORI-*perR* versus empty vector pORI23). Taken together, these data provide the first direct evidence that PerR-regulated gene expression is required for optimal GAS resistance to phagocytic killing during infection.

**Table 1:** Resistance to phagocytic killing of wild-type and *perR* mutant GAS in human blood.

Strain	Cfu^a^ (0 h)	Cfu^a^ (3 h)	Fold Increase^a^ Cfu 3 h/ Cfu 0 h
003Sm	1.16±0.20×10^4^	4.65±2.15×10^4^	4.0±1.8
003Sm*perR*Δ	1.18±0.21×10^4^	1.23±0.53×10^4 ^ b	1.0±0.4

a Mean±standard deviation of 4 independent experiments.

b *P*<0.03 for comparison of 003Sm*perR*Δ versus 003Sm cfu at 3 h (Student's *t*-test).

### PerR-regulated gene expression counteracts GAS killing by the macrophage oxidative burst

**Figure 2:**
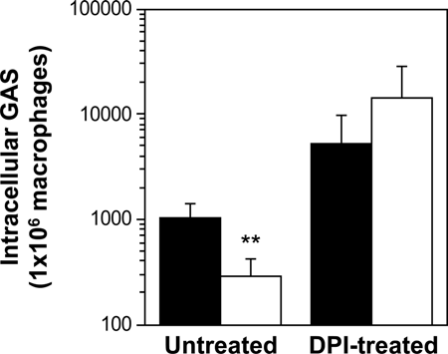
Attenuated survival of the GAS *perR* mutant inside macrophages is rescued by inhibition of the macrophage oxidative burst. Mouse RAW246.7 macrophages were infected for 1 h with wild-type strain 003Sm (black bars) or *perR* mutant strain 003Sm*perR*Δ (white bars) and then treated with penicillin-gentamicin for an additional hour in the absence (untreated) or presence (DPI-treated, 10 µM) of the general respiratory burst inhibitor DPI. Following the 1 h treatment, intracellular GAS were recovered and enumerated by quantitative culture. Note that DPI treatment rescues attenuated survival of the *perR* mutant evident in the absence of the inhibitor. **p<0.04 for comparison of 003Sm*perR*Δ versus 003Sm cfu (Student's *t*-test).

The mechanism by which PerR contributes to GAS survival inside phagocytic cells was further investigated using a mouse macrophage cell line. Wild-type strain 003Sm or *perR* mutant 003Sm*perR*Δ was used to infect RAW264.7 macrophages and the ability of the mutant to survive intracellularly was compared to that of wild-type bacteria. Intracellular survival was measured in the absence or presence of diphenyleneiodonium chloride (DPI), an inhibitor of both NADPH oxidase and nitric oxide synthase, in order to investigate the effect of the macrophage oxidative burst on both wild-type and mutant GAS. In the absence of DPI, survival of the mutant inside macrophages was 3- to 4-fold lower than that of wild-type, as indicated by the number of intracellular bacteria recovered from infected macrophages during gentamicin/penicillin exclusion assays (Figure 2). This survival attenuation of the mutant inside macrophages was in agreement with reduced resistance to phagocytic killing in human blood shown above. DPI treatment of infected macrophages resulted in an approximate 10-fold increase in the number of viable intracellular wild-type bacteria. Whereas the number of intracellular *perR* mutant bacteria recovered from untreated macrophages was significantly lower than that of wild-type, in the presence of DPI intracellular survival of the mutant was similar to wild-type GAS (Figure 2). The finding that the *perR* mutant is hypersensitive to macrophage killing and that its intracellular survival defect is abrogated by DPI suggests that GAS killing by the macrophage oxidative burst is counteracted, at least partially, by PerR-dependent gene expression.

### PerR is required for GAS pharyngeal colonization

**Figure 3:**
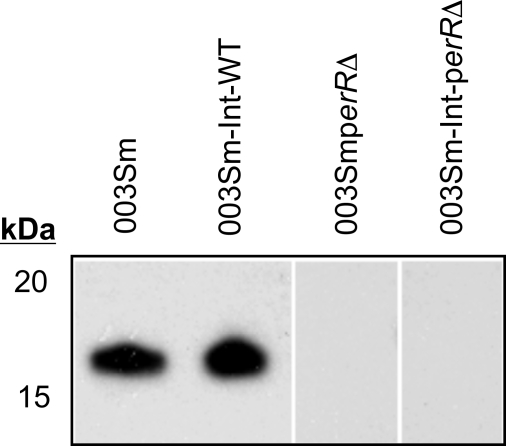
PerR expression in GAS integrant strains 003Sm-Int-*perR*Δ and 003Sm-Int-WT and the *perR* deletion mutant strain 003Sm*perR*Δ as determined by immunoblotting. Cell lysates of the three strains, as well as of parent strain 003Sm, were fractionated by SDS-PAGE and immunoblotted with PerR-specific rabbit antiserum. Note the loss of PerR in both 003Sm*perR*Δand 003Sm-Int-*perR*Δ in contrast to wild-type PerR expression levels in 003Sm-Int-WT.

A major goal of this study was to define the contribution of PerR in GAS survival and persistence in the pharynx, the most commonly infected human host site. To achieve this goal, we used a baboon pharyngeal colonization model that recapitulates several aspects of GAS human infection [20]. Quantitative throat cultures were used to assess pharyngeal colonization by wild-type strain 003Sm compared with that of the *perR* mutant integrant strain 003Sm-Int-*perR*Δ in five baboons co-infected with approximately 1.5×10^8^ cfu of each of the two strains. Co-infection with parent strain 003Sm served as an internal reference in each animal and was adopted to control for the variability in GAS pharyngeal colonization levels previously observed among different animals. Expression of *perR* in strain 003Sm-Int-*perR*Δ is abolished by the stable chromosomal integration of plasmid pJL*perR*Δ (Figure 1) that also confers chloramphenicol (Cm) resistance, a marker that was exploited to differentiate mutant strain 003Sm-Int-*perR*Δ from the Cm-sensitive parent strain. In addition, to control for possible nonspecific attenuating effects of plasmid insertion in the GAS chromosome or potential excision of the plasmid from the chromosome during infection, a second group of five animals was co-infected with similar doses of 003Sm and integrant strain 003Sm-Int-WT in which pJL*perR*Δ integration had occurred immediately downstream, and not upstream, of *perR* (Figure 1). As predicted by their respective genotypes, strain 003Sm-Int-*perR*Δ did not express PerR and was hyper-resistant to H_2_O_2_ challenge similarly to the deletion mutant strain 003Sm*perR*Δ while strain 003Sm-Int-WT produced wild-type amounts of PerR, as shown in immunoblots using PerR-specific antiserum (Figure 3), and exhibited similar H_2_O_2_-sensitivity as the wild-type strain (data not shown).

**Figure 4:**
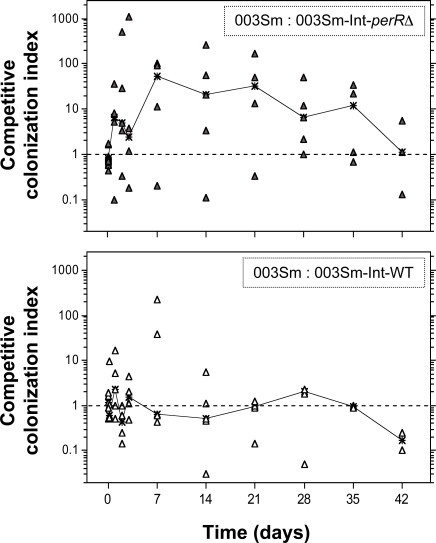
Competitive pharyngeal colonization in baboons following co-infection with wild-type GAS strain 003Sm and PerR mutant 003Sm-Int-*perR*Δ (top panel), or with 003Sm and PerR-expressing strain 003Sm-Int-WT (bottom panel). Symbols represent competitive colonization indices (CCIs) calculated for each infected animal at each of eleven time points. Black lines indicate the median CCI values in the two groups of infected animals over time. CCI values are shown for samples that yielded at least one of two co-infecting strains. A CCI equal to 1, indicated by a broken horizontal line in both panels, represents identical counts for the two co-infecting strains.

All 10 animals were infected by direct pipeting of pre-mixed suspensions of one of the two strain pairs onto the posterior pharynx (003Sm/003Sm-Int-*perR*Δ or 003Sm/003Sm-Int-WT). A total of 11 sequential throat swabs were collected from each animal at various time points up to 42 d post-inoculation for quantitative culture on both Sm and Cm plates. Relative colonization of wild-type versus each of the two integrant strains was calculated by dividing the wild-type cfu count by the cfu count of the plasmid integrant strain studied, and was represented as the competitive colonization index (CCI) at each time point for each animal. All baboons were colonized for 28 d or longer except for one animal in the control group (003Sm/003Sm-Int-WT) that was colonized for 14 d (Figure S2). At 1 and 3 h post-infection, the GAS counts in both groups were reduced drastically compared to the inoculum; however, the counts of each of the two integrant strains were similar to those of the wild-type parent strain, as indicated by CCIs of approximately 1 in nine of 10 animals (Figure 4). A further decrease in colonization occurred in most animals at 24 to 48 h (Figure S2), a pattern similar to that observed in previous primate studies of GAS throat colonization [20]–[22]. Interestingly, the 003Sm/003Sm-Int-*perR*Δ median CCI increased from approximately 1 at 3 h to 4.8 at 48 h post-infection, whereas the median CCI in the control group (003Sm/003Sm-Int-WT) was approximately 11-fold lower at 0.43. Following the initial drop, colonization levels increased in most animals of both groups after day 3. In the control group, the two strains were recovered in similar relative numbers throughout the experiment (Figure S2), as indicated by median CCIs of approximately 1 (Figure 4). In contrast, the median CCI in the 003Sm/003Sm-Int-*perR*Δ infected group increased further after day 3 reaching values of 53, 21, and 32 on days 7, 14, and 21, respectively, which were 33- to 81-fold higher than those recorded for the control group at respective time points (Figure 4).

**Figure 5:**
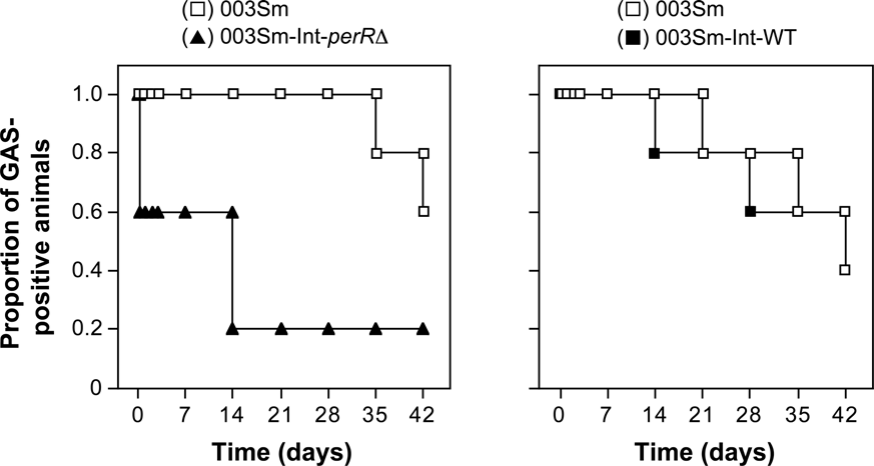
Time-to-clearance of wild-type strain 003Sm versus PerR mutant strain 003Sm-Int-*perR*Δ (left panel) or 003Sm versus PerR-expressing strain 003Sm-Int-WT (right panel) in two groups of five co-infected baboons, respectively. The proportion of animals that yielded positive throat cultures for each of the two co-infecting strains in each group is shown. In the left panel, note the rapid clearance of the *perR* mutant strain 003Sm-Int-*perR*Δ (closed triangles) versus the persistence of the wild-type parent strain (open squares) in the same animals. In the control group shown in the right panel, persistence of the PerR-expressing strain 003Sm-Int-WT (closed squares) was similar to that of the wild-type strain 003Sm (open squares).

The competitive infection experiments were analyzed also as time-to-clearance curves, that is, the time to persistently negative cultures for each of the three strains used. This analysis showed that the *perR* mutant strain 003Sm-Int-*perR*Δ was cleared by two of five baboons within hours of infection and was not recovered from four of five animals after day 7 (Figure 5). In sharp contrast, the wild-type strain colonized all five animals in the same group for at least 28 d, with three of five animals staying GAS-positive for the duration of the study (42 d). As expected, in the control animals the time-to-clearance of the control integrant strain 003Sm-Int-WT did not differ from that of wild-type strain 003Sm. Together, these data demonstrate that the *perR* mutant strain 003Sm-Int-*perR*Δ is severely attenuated in its capacity to colonize the baboon pharynx. The rapid elimination of the mutant from the baboon throat underlines the importance of PerR in virulence and suggests a critical role of PerR-regulated gene expression in GAS survival and persistence in the pharynx during human infection.

### PerR controls multiple loci with diverse functions in strain 003Sm

**Figure 6:**
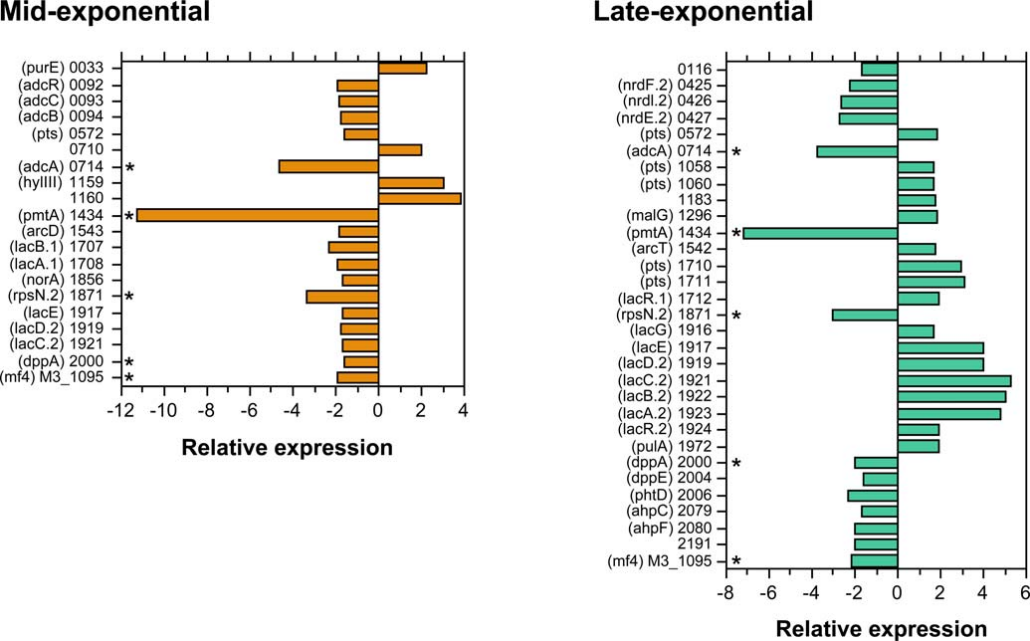
Genes controlled by PerR in GAS strain 003Sm during growth in mid-exponential (A_600nm_ = 0.25) or late-exponential (A_600nm_ = 0.60) phase growth. Values represent relative expression levels (fold-change) in wild-type bacteria compared to those in the *perR* mutant strain 003Sm*perR*Δ. Numbers on *y*-axis indicate SPy or spyM3 ORF numbers corresponding to M-type 1 strain SF370 [32] and M-type 3 GAS strain MGAS315 [18], respectively. *Genes regulated in similar fashion at both growth phases.

To gain insight into the gene network controlled by PerR in strain 003Sm and to identify candidate loci contributing to GAS pharyngeal infection and virulence, we compared the transcriptome of mutant strain 003Sm*perR*Δ with that of 003Sm using GAS genomic microarrays. Total RNA was isolated from both strains grown to mid- or late-exponential phase and mRNA was converted to fluorescently labeled cDNA and hybridized to the GAS microarray. A total of 42 genes, including five encoding hypothetical proteins, were differentially regulated in wild-type bacteria: 20 of these were regulated at mid-exponential phase and 31 at late-exponential phase growth (Figure 6). Five genes (*SPy0714, SPy1434*, *SPy1871*, *SPy2000*, *spyM3_1095*) showed similar regulation in both growth phases and were among the most highly downregulated loci. Unexpectedly, 21 of the 42 PerR-dependent genes exhibited higher expression levels in wild-type bacteria, a finding that suggests PerR may act, directly or indirectly, as an activator, as well as a repressor. Sixteen of these 21 genes were identified during late-exponential phase growth, an indication that PerR-dependent gene activation is growth phase-dependent and may involve specific physiological conditions and/or additional factors absent during early exponential phase (Figure 6).

The microarray data were validated by quantitative RT-PCR (qRT-PCR) using total RNA isolated from three independent cultures of wild-type and *perR* mutant bacteria in each of two growth phases. Based on the data obtained for a total of 17 genes representing the majority of PerR-dependent loci (Table 2), there was strong correlation between qRT-PCR and microarray data analyses (*r* = 0.89). PerR regulation seemed to be somewhat variable for three of these genes (*SPy0033*, *SPy1159*, *SPy1707*), as their regulation, though confirmed in the RNA preparations used for microarray studies (*r* = 0.98), could not be validated in independent RNA preparations.

**Table 2:** qRT-PCR validation of PerR-dependent genes.

			Relative expression^c^	
SPy Number^a^	Gene	Function^b^	Microarray	qRT-PCR
*Mid-exponential*				
0033 (0027)	*purE*	Phosphoribosylaminoimidazole carboxylase catalytic subunit	+2.2	+1.2 (0.1)
0092 (0069)	*adcR*	Putative repressor protein	−1.9	−1.3 (0.3)
0710 (1208)		Conserved hypothetical protein	+2.0	+2.0 (0.5)
0714 (0466)	*adcA*	Putative adhesin (zinc-binding)	−4.6	−3.2 (0.2)
1159 (0815)	*hylIII*	Putative hemolysin III	+3.0	−1.2 (0.4)
1434 (1093)	*pmtA*	Putative metal transport ATPase	−11.3	−15.8 (3.6)
1707 (1484)	*lacB.1*	Galactose 6-phosphate isomerase	−2.3	+1.1 (0.2)
1871 (1615)	*rpsN.2*	30S subunit ribosomal protein S14	−3.3	−2.5 (0.5)
*Late-exponential*				
0426 (0302)	*nrdI.2*	Putative ribonucleotide reductase	−2.7	−1.5 (0.4)
1710 (1487)	*pts*	Putative PTS enzyme IIB	+2.9	+5.9 (2.1)
1712 (1489)	*lacR.1*	Putative lactose PTS repressor	+1.9	+2.5 (0.8)
1917 (1654)	*lacE*	Putative PTS enzyme IIBC	+4.0	+10.7 (0.8)
1922 (1658)	*lacB.2*	Galactose 6-phosphate isomerase	+5.0	+6.6 (2.3)
1924 (1660)	*lacR.2*	Putative lactose PTS repressor	+1.9	+2.2 (0.6)
2006 (1724)	*phtD*	Hypothetical protein	−2.3	−5.6 (2.1)
2079 (1770)	*ahpC*	Putative alkyl hydroperoxidase	−1.7	−1.9 (0.3)
n/a^d^ (1095)	*mf4*	Putative mitogenic factor/DNase	−2.2	−2.1 (0.4)

a SPy ORF number of M-type 1 strain SF370 [32]; spyM3 ORF number of M-type 3 strain MGAS315 [18] in parentheses.

b NCBI annotation.

c Fold-change (standard deviation) of expression in wild-type strain 003Sm compared to expression in *perR* mutant 003Sm*perR*Δ.

d Non-applicable, *spyM3_1095* only present in M-type 3 GAS.

**Figure 7:**
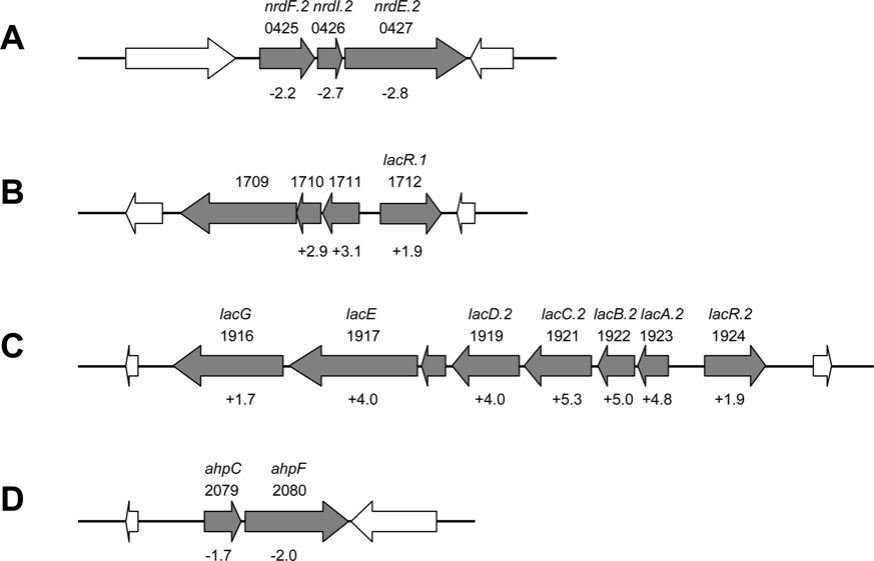
Genetic organization of four PerR-dependent predicted operons in strain 003Sm. The ribonucleotide reductase system (A), the putative lactose/galactose PTSs SPy1709-SPy1711 (B) and SPy1923-SPy1916 (C) with their putative cognate regulators in opposite orientation, and the alkyl hydroperoxidase/alkyl hydroperoxide reductase AhpCF (D) loci are shown. The genes and ORF numbers for each locus in M-type 1 GAS strain SF370 [32] are indicated. Numbers below the genes indicate relative expression levels (fold-change) in wild-type strain 003Sm compared to *perR* mutant strain 003Sm*perR*Δ recorded in the microarray experiments.

Several PerR-dependent genes could be associated with GAS oxidative stress adaptation responses and virulence (Table S1). Among these, *pmtA* (*SPy1434*), the most highly PerR-repressed gene identified, encodes a metal transport ATPase recently shown to be under PerR control in M-type 5 GAS. In that study, PmtA was suggested to contribute to H_2_O_2_ resistance *in vitro* but its role *in vivo* remains unclear [16]. Also, among these genes was *SPy2079*-*SPy2080* encoding alkyl hydroperoxidase and NADH oxidase/alkyl hydroperoxide reductase (AhpC and AhpF, respectively) (Figure 7). AhpCF is thought to catalyze the NADH-dependent reduction of peroxides, and has been shown to contribute to GAS oxidative stress resistance and virulence in mice [1],[2],[4]. Although regulation of this locus was not strong, at least under the conditions tested, AhpCF is the first GAS peroxidase system found to be PerR-dependent. In addition, expression of the ribonucleotide reductase operon *nrdF.2-nrdI.2-nrdE.2* (*SPy0425*-*SPy0427*) was PerR-dependent (Figure 7). Ribonucleotide reductases are required for the conversion of ribonucleotides to deoxyribonucleotides and may be critical for DNA replication under oxidative stress for repair of DNA damage by ROS [23]–[25]. The M-type 3-specific prophage gene *spyM3_1095*, encoding DNase/mitogenic factor 4 (Mf4) was also included in this group. Expression of this extracellular DNase was shown to be activated under oxidative stress, as well as following DNA damage in M-type 3 strain MGAS315 [26]. Finally, *SPy0714* encoding the putative adhesin AdcA was strongly regulated at both growth phases studied.

It was striking that 40% (17 of 42) of PerR-dependent loci are predicted to encode products involved in sugar metabolism and/or transport, with the majority being upregulated by PerR (directly or indirectly) during late-exponential phase growth. These loci included two phosphotransferase systems (PTSs), SPy1710-SPy1711 and SPy1916-SPy1923 (*lacA.2*-*lacG*), with putative function in lactose/galactose utilization [27],[28]. Moreover, a PTS transcriptional regulator that exhibited similar PerR-dependent up-regulation mapped immediately upstream and in the opposite orientation of each of these two loci (SPy1712, SPy1924), an indication that PerR regulation of these two PTS loci might be indirect through control of their respective cognate regulators (Figure 7). These findings suggest an important role of PerR in sugar uptake and utilization, as well as oxidative stress adaptive responses of GAS.

In summary, 35 of a total of 42 genes shown to be PerR-dependent in strain 003Sm were not previously considered to be under PerR control. These included genes mainly regulated during late-exponential phase growth, such as most PTS/sugar metabolism genes, as well as genes with putative function in oxidative stress responses and virulence. These findings suggest that PerR controls, directly or indirectly, a more extensive and functionally more diverse regulon than previously reported in GAS.

### Sequence analysis and interaction of PerR with promoters of PerR-dependent loci

In order to investigate whether the PerR-dependent genes identified in microarray studies were directly regulated by PerR in strain 003Sm, the M-type 3 strain MGAS315 genome [18] was scanned for the previously proposed GAS Per box operator sequence [4]. This analysis revealed canonical Per boxes upstream of *pmtA* and *ahpCF*, which is in agreement with earlier reports of PerR-regulated gene expression in GAS [4],[16]. Additional Per box-like promoter sequences were identified upstream of several PerR-dependent loci in strain 003Sm, such as *nrdF.2-nrdI.2-nrdE.2*, PTSs *SPy1710*-*SPy1711* and *SPy1916-SPy1923* (*lacA.2-lacG*) as well as their cognate regulators *lacR.1* and *lacR.2* (Table 3). A non-canonical Per box sequence has been previously reported for the DNA-binding peroxide resistance gene *mrgA* also thought to be PerR-regulated in GAS, although PerR binding to the *mrgA* promoter has not been shown [4].

**Table 3:** Per box promoter sequences of PerR-dependent loci in strain 003Sm.

GAS Locus	Promoter Sequence	Distance from Start (nt)^a^
*ahpC*	TTAGAATCATTTTAA	139
*lacA.2*	ATTTACTTATTCTAA	16
*lacR.2*	TTAT GATACCTTTAA	34
*nrdF.2*	TTAT GGTTTTTATAA	257
*pmtA*	TTAGAATTATTATAA	49
*rpsN.2*	GTATAATGATTATAT	88
*SPy1711*	CTATAATGGTTTTGT	72
*lacR.1* (SPy1712)	ACAAAACCATTATAG b	149
Consensus^c^	TTANAATNATTNTAA	

a Number of nucleotides (nt) between the 3′-end of Per box sequence and the predicted start codon of indicated gene in M-type 3 GAS strain MGAS315 [18].

b Reverse complement of Per box-like sequence upstream of *SPy1711.*

c 15 bp Per box consensus [4]; promoter sequences matching with consensus underlined.

Since several of the differentially regulated loci in the M-type 5 *perR* mutant had been previously suggested to be under AdcR control [16], with their differential regulation a secondary effect of *pmtA* upregulation in the mutant, a second M-type 3 genome search was performed for AdcR consensus sequences [29]. AdcR motifs were identified upstream of four loci differentially regulated in *perR* mutant strain 003Sm*perR*D: *adcRCB* (*SPy0092*-*SPy0094*), *adcA* (*SPy0714*), *rpsN.2* (*SPy1871*) and *phtD* (*SPy2006*). Our results of AdcR motif search in M-type 3 GAS are in line with those reported by Brenot et al. [16] and imply that regulation of the latter loci is also under AdcR control in strain 003Sm.

**Figure 8:**
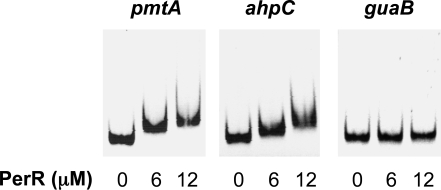
PerR binds to *pmtA* (*spyM3_1093* or *SPy1434*) and *ahpC* (*spyM3_1770* or *SPy2079*) promoters (242 bp and 219 bp, respectively) encompassing canonical Per boxes. Each of the two promoter fragments, or the negative control *guaB* promoter (246 bp), was incubated with recombinant his_6_-PerR at the concentrations indicated for 15 min at room temperature. Following analysis on native polyacrylamide gels, electrophoretic mobility shift of each promoter fragment was visualized by ethidium bromide staining.

Following *in silico* analyses, PerR binding to promoters encompassing predicted Per boxes was tested by electrophoretic mobility shift assays (EMSAs). Recombinant PerR was expressed in *E. coli* as an N-terminal his-tagged protein and was purified under native conditions. Promoters tested in EMSAs included those of the putative metal transport ATPase *pmtA* and the alkyl hydroperoxidase system *ahpCF*, both of which include canonical Per boxes, the promoter upstream of the PTS *SPy1916-1923* (*lacA.2-lacG*) as well as its divergently transcribed putative cognate regulator *lacR.2* (SPy1924), and the promoter sequence in the intergenic region upstream of PTS *SPy1710-1711* and its putative cognate regulator gene *lacR.1* (*SPy1712*) that contains a common Per box (Table 3). As shown in Figure 8, recombinant PerR bound to both *pmtA* and *ahpC* promoters but did not bind to promoters encompassing non-canonical Per boxes, such as those upstream of the PTS loci (data not shown). It is possible that PerR binding to the latter promoters requires different conditions from those tested and/or additional factors that may be present in GAS *in vivo*. Alternatively, these genes may not be directly regulated by PerR, with their altered expression in mutant strain 003Sm*perR*Δ being a secondary effect of PerR deletion.

## Discussion

The present investigation provides direct new evidence that PerR plays a key role in GAS adaptation for survival in the human host and uncovers a mechanism through which it contributes to GAS virulence. PerR-dependent gene expression was associated with enhanced resistance to ROS-dependent phagocytic killing by macrophages *in vitro* and with improved survival in human blood. The PerR system also enhanced GAS pharyngeal colonization in a primate model that mimics GAS pharyngitis in human beings [20]. In this model, wild-type GAS showed a clear competitive advantage over a *perR* mutant in both its ability to maintain high colonization levels and to persist in the pharynx over time. The marked attenuation of the *perR* mutant is similar to that reported in the baboon model for GAS mutants lacking the hyaluronic acid capsule or M protein, two of the most important virulence determinants of the species [20]. To our knowledge, this is the first study to demonstrate the key role of PerR-dependent gene expression in GAS pharyngeal infection and to directly associate PerR with phagocytic killing resistance in human blood.

Our comparative analysis of global gene expression in M-type 3 strain 003Sm versus its isogenic *perR* mutant revealed a PerR-dependent regulon of 42 genes. Although some of these had been reported earlier [16], the majority of the PerR-dependent loci in strain 003Sm were not previously identified. Discovery of new PerR-dependent genes in the present study may reflect the fact that many were identified in late-exponential phase samples, whereas earlier investigations compared wild-type to *perR* mutant transcriptomes during mid-exponential phase. It is likely that some of the differences reported also reflect strain-dependent variation among GAS isolates. For instance, expression of both superoxide dismutase (*sodA*; *SPy1406*) and the metal transport system MtsABC (*SPy0453*-*SPy0455*) was moderately affected in an M-type 1 GAS *perR* mutant [6], but no such evidence was found in this or other studies involving GAS strains of M-type 1, 3, and 5 [15],[16]. In addition, the Dps-like peroxide resistance gene *mrgA* (*SPy1531*), the putative cold shock protein gene *csp* (*SPy2077*), and the cation transport gene *czcD* (*SPy0845*) previously shown to be under PerR control in M-type 5 GAS were not identified in the present study [4],[16].

Several of the PerR-regulated gene products have been implicated in GAS oxidative stress responses and resistance mechanisms. The metal transporter PmtA has been linked to H_2_O_2_ resistance *in vitro* but its role in virulence remains unclear, as low *pmtA* expression was observed in GAS recovered from mouse skin ulcers [16]. Nonetheless, the strong regulation of *pmtA* by PerR in both the M-type 5 and M-type 3 strain backgrounds argues for an important role of this metal transporter in GAS physiology and perhaps virulence. Increased metal efflux due to *pmtA* upregulation has been suggested to lead to H_2_O_2_ hyper-resistance of the *perR* mutant during growth in metal-replete conditions *in vitro* [16]. In the metal-deplete host environment, however, PmtA upregulation could lead to metal starvation that might be in part the reason for reduced fitness and virulence of the *perR* mutant during infection. Thus, coordinate control of PmtA expression by PerR might vary depending on the host environment encountered. The role of the alkyl hydroperoxidase system (AhpCF) in oxidative stress resistance and virulence of GAS has been demonstrated [1],[2],[4]. Although the degree of *ahpCF* regulation by PerR was only moderate (1.7- to 2.0-fold) under the experimental conditions of this study, such regulation may still have a significant effect on oxidative stress resistance of GAS. Initial studies suggest 2- to 3-fold upregulation of *ahpC* following GAS challenge with H_2_O_2_ during culture *in vitro*; however, additional microarray studies with peroxide and perhaps other stress agents (oxidative and non-) will be essential to identify the physiological environmental stimulus for PerR and the resulting PerR-mediated regulation events in GAS. PerR-dependent expression was also demonstrated for the M-type 3 specific phage-encoded DNase Mf4 (*spyM3_1095*) that was previously shown to be induced under oxidative stress and DNA damage [26]. DNases (phage-encoded and non-) have been shown to enhance extracellular phagocytic killing resistance of GAS by degrading human neutrophil extracellular traps [30],[31]. A similar function in phagocytic killing resistance of M-type 3 GAS is possible for Mf4.

Interestingly, expression of the ribonucleotide reductase operon *nrdF.2-nrdI.2-nrdE.2* was also PerR-dependent. Ribonucleotide reductase systems catalyze deoxyribonucleotide synthesis which is essential for DNA replication and repair. Such fundamental function is critical for bacterial survival and may be required for DNA replication under all growth conditions. Upregulation of this operon could also allow repair of DNA damage due to ROS under oxidative conditions. A second ribonucleotide reductase system encoded by *spyM3_1048*-*spyM3_1050* and *spyM3_1706*, representing NrdH-NrdE.1-NrdF.1 and NrdI.1, respectively, is found in both M-type 1 and 3 GAS genomes [18],[32]. While the *nrdIEF* locus seems to be essential for growth of *B. subtilis* [23],[25], an absolute requirement of one or both ribonucleotide reductase systems in GAS has not been established.

Approximately half of the PerR-dependent genes in strain 003Sm are activated by PerR (directly or indirectly), and a significant proportion of these are predicted to encode products involved in sugar metabolism and transport. This result suggests a novel role for PerR in regulating sugar uptake and utilization by GAS. PerR activation (direct or indirect) of PTSs and/or other sugar utilization pathways may reflect the increased energy utilization required to counteract cell toxicity inflicted upon the bacteria by the inflammatory response. Utilization of multiple sugar sources during infection is likely to increase bacterial survival and could be an important contribution of PerR-regulated gene expression to GAS virulence. The dramatically attenuated colonization capacity of the *perR* mutant in the baboon model could be the result of poor uptake and/or utilization of available sugar sources in the oropharynx together with reduced resistance to phagocytic killing. Recent evidence shows that GAS utilization of maltodextrins is specifically upregulated in human saliva, an event that has been proposed to contribute to GAS survival in saliva and to efficient colonization of the mouse oropharynx [33]. Currently, whether any of the PerR-dependent PTSs contributes to GAS fitness in the host remains unknown.

In *B. subtilis*, PerR represses target gene expression by binding to conserved Per box promoter sequences. Bound PerR is released from promoters under oxidative conditions upon its auto-oxidation by a regulatory ferrous ion [9]. The PerR regulation mechanism in GAS may be similar, as purified recombinant PerR contains both zinc and ferrous ions (I. Gryllos and D. Kurtz Jr; unpublished observations). In addition, a Per box consensus sequence similar to that of *Bacillus* has been proposed for GAS; however, except for *pmtA* and *ahpCF*, this sequence is not fully conserved in promoters thought to be controlled by PerR [4],[16]. PerR binding to a canonical Per box upstream of *ahpCF* was reported previously in GAS, but PerR-dependent regulation of this locus was very weak, if any, at mid-exponential phase growth [4]. In the present study, moderate regulation of *ahpCF* was reported during late-exponential phase growth with weaker regulation observed during mid-exponential phase growth. Binding of recombinant PerR to both *pmtA* and *ahpCF* promoters in electrophoretic mobility shift assays confirms direct PerR regulation of the *ahpCF* locus and also demonstrates direct regulation of *pmtA*. In addition, Per box-like sequences were identified upstream of several other PerR-dependent loci in strain 003Sm, of which some were predicted to be activated, and not represssed, by PerR. Unlike the promoters encompassing fully conserved Per boxes, the promoters of the PerR-activated PTS loci did not bind recombinant PerR *in vitro*. Interestingly, the majority of gene activation events were observed in late-exponential phase growth, which suggests that specific conditions and/or additional factors may be required for PerR regulation at these promoter sites. At present, whether additional factors affect PerR regulation of certain promoters by interacting with the promoter sequence, with PerR, or both, remains unclear. Nonetheless, the presence of Per box-like sequences upstream of loci that require PerR for activation implies a potential PerR interaction with Per box sequences during gene activation as well as repression. Ferrous ion-mediated PerR oxidation resulting in a conformational change could be part of an activation mechanism. PerR is also thought to be required for maximal expression of several genes in *B. subtilis*, and direct PerR gene activation through binding to two tandemly arranged non-canonical Per boxes has been reported for the surfactin (*srfA*) operon [11],[34]. Initially considered to be exclusively a repressor, the prototype of the PerR family, Fur, has been suggested to also activate gene expression [35],[36]. Currently, the possibility that PerR does not activate gene expression in GAS through direct interaction with specific promoters, but instead *perR* deletion leads to indirect effects in sugar utilization and other loci cannot be excluded.

Taken together, our data show a pivotal role of PerR-controlled gene expression in GAS resistance to phagocytic killing and a critical contribution to host adaptation for survival in the pharynx. In addition, the evidence presented expands significantly on our knowledge of the PerR regulon in GAS. The diverse functions of newly identified PerR-dependent loci suggest a previously unrecognized role of PerR that may contribute to increased bacterial fitness in the host. Future investigations into the contribution of these loci to GAS virulence, as well as the molecular events governing PerR gene regulation, should further elucidate PerR function during human infection.

## Materials and Methods

### Bacterial strains and growth conditions

GAS strain DLS003 is an M-type 3 strain isolated from a patient with necrotizing fasciitis [17]. GAS was grown at 37°C in Todd-Hewitt broth (Difco Laboratories) supplemented with 0.5% yeast extract (THY), or on THY agar or trypticase-soy agar, both supplemented with 5% defibrinated sheep blood. For cloning experiments, Escherichia coli DH5α or XL1-blue was grown in Luria-Bertani (LB) broth (Difco Laboratories) or on LB agar. When appropriate, antibiotics were added at the following concentrations: ampicillin 100 µg/ml; chloramphenicol 20 µg/ml for E. coli, 10 µg/ml for GAS; erythromycin 300 µg/ml for E. coli, 0.5 µg/ml for GAS; gentamicin 200 µg/ml; kanamycin 30 µg/ml; penicillin 20 µg/ml; streptomycin 200 µg/ml.

### DNA and RNA techniques

Plasmid pJL1055 is a temperature-sensitive *E. coli*–Gram-positive shuttle vector previously used for allelic replacement mutagenesis in group B *Streptococcus* [37]; pORI23 is an *E.coli*–Gram-positive shuttle vector previously used for complementation studies in GAS [38],[39]. Restriction endonuclease digestions, DNA ligations and transformations of chemically competent *E. coli* were performed using standard protocols [40]. GAS chromosomal DNA isolation and GAS electroporations were performed as described [41],[42]. Total GAS RNA was isolated using the RNeasy mini kit (Qiagen) from bacterial lysates obtained by shaking with glass beads on a dental amalgamator [43].

### PCR, reverse transcriptase PCR (RT-PCR), and quantitative RT-PCR (qRT-PCR)

Primers used were designed based on the M-type 3 GAS strain MGAS315 genome sequence [18] (Table S2). PCR was performed with *Taq* Platinum DNA polymerase (Invitrogen). Semi-quantitative RT-PCR was performed using the Access RT-PCR system (Promega) for 20–30 amplification cycles and 30 ng total bacterial RNA template. qRT-PCR was performed on an ABI PRISM 7000 Sequence Detection System (Applied Biosystems) using the QuantiTect SYBR green RT-PCR kit (Qiagen) as described [39]. Expression levels of each test gene were normalized to those of *recA* (*spyM3_1800*), which did not show any change in expression as a consequence of *perR* mutation. Data were reported as mean relative expression levels (±standard deviation) in wild-type versus *perR* mutant.

### Construction of a *perR* deletion mutant in strain 003Sm and complementation

The *perR* deletion mutant was derived from strain 003Sm, a spontaneous streptomycin resistant (Sm^R^) variant of wild-type strain DLS003. Sm resistance was required for baboon throat colonization experiments (see below). Wild-type *perR* (*spyM3_0147*) on 003Sm chromosome was exchanged with a truncated *perR* copy by allelic exchange as described [43]. Approximately 500 bp of *perR* upstream and downstream flanking sequence encompassing approximately 20 bp of the 5′-end and the 3′-end of *perR*, respectively, was obtained by PCR using the 003Sm chromosome as template and primer pairs spyM3_0146-F/perR-R and perR-F/spyM3_0148-R, respectively. The forward primer binding to the 3′-end of *perR* (perR-F) was designed to carry complementary sequence to the reverse primer binding to the 5′-end of the gene (perR-R). The overlapping sequence incorporated in the two PCR products allowed fusion of the two fragments in a subsequent PCR by using both fragments as template and the two outside primers (spyM3_0146-F and spyM3_0148-R) for amplification. The resulting fragment carrying the truncated *perR* copy was cloned into the temperature-sensitive *E. coli*-Gram-positive shuttle vector pJL1055 to create plasmid pjL*perRΔ.* The *perR*Δ construct was introduced into 003Sm by electroporation and crossed into the chromosome by homologous recombination with subsequent excision of the wild-type copy as described [43]. The *perR* deletion mutant strain 003Sm*perR*Δ was obtained from integrant strain 003Sm-Int-*perR*Δ following several passages at 30°C in the absence of antibiotics in order to promote pJL*perR*Δ excision from the chromosome. The chromosomal deletion and the lack of *perR* specific mRNA in strain 003Sm*perR*Δ were confirmed by PCR and reverse transcriptase PCR (RT-PCR), respectively. PCR and qRT-PCR amplification of *perR* flanking genes (*spyM3_0146* and *spyM3_0148*) yielded identical results in wild-type and mutant bacteria indicating no upstream or downstream effects during *perR* mutagenesis. Loss of PerR expression in the mutant was confirmed by immunoblotting of mutant protein extracts with PerR antiserum raised against purified recombinant PerR.

For *perR* complementation, the *perR* coding sequence including the predicted ribosomal binding site was amplified from the wild-type strain 003Sm chromosome using primer pair perR-F-RBS (BamHI)/perR-R (PstI) and was cloned into BamHI/PstI-digested shuttle vector pORI23. The resulting plasmid pORI-*perR* was introduced into *perR* mutant strain 003Sm*perR*Δ by electroporation. PerR expression *in trans* was confirmed by immunoblotting of cell lysates of 003Sm*perR*Δ (pORI-*perR*), or 003Sm*perR*Δ (pORI23) as control, using PerR specific antiserum. These studies showed significantly higher PerR expression level in strain 003Sm*perR*Δ (pORI-*perR*) than in the wild-type strain 003Sm (data not shown).

### Purification of recombinant PerR and antiserum production

A 465 bp fragment encoding PerR without its start codon was amplified by PCR from GAS strain 003Sm chromosome using primers perR-F (BamHI) and perR-R (HindIII). The product was cloned into BamHI/HindIII-digested pQE30 (Qiagen) downstream of the RGS-his_6_ tag sequence, and expression of the 19 kDa his-tagged recombinant peptide was performed in *E. coli* strain M15 (pREP4) using IPTG induction (1 mM final concentration). Purification from *E. coli* lysates was achieved by Ni^2+^-affinity chromatography using Ni-NTA resin (Qiagen) under native conditions. The purified recombinant peptide was used to immunize rabbits for PerR antiserum production.

### GAS protein preparation and immunoblotting

GAS protein preparations were obtained from 10 ml cultures grown to mid-exponential growth phase as described [39]. Preparations were analyzed by SDS-PAGE under reducing conditions using 10% NuPAGE Bis-Tris gels (Invitrogen). For immunoblotting, proteins were transferred onto a nitrocellulose membrane and blocked in PBS-5% milk (Difco Laboratories). Anti-PerR rabbit antiserum was added at 1∶5000 in PBS-5% milk for 1 h at room temperature followed by 1 h incubation in peroxidase-conjugated secondary antibody (GE Healthcare). Membrane development was performed using the ECL detection kit (GE Healthcare).

### H_2_O_2_ killing assays

Resistance of GAS to oxidative killing by H_2_O_2_ was tested as described [6] with slight modifications. Bacteria were grown overnight (16 h) in 11 ml THY broth in sealed culture tubes and then sub-cultured in 10 ml broth at a starting density of A_600nm = _0.05. Cultures were grown for approximately 2 h to early exponential phase (A_600nm_ = 0.15) at which point they were challenged with H_2_O_2_ at a final concentration of 10 mM. Following 1 h incubation with H_2_O_2_ at 37°C, culture samples were removed for quantitative GAS culture on tryptic soy-blood agar under aerobic conditions. Percent survival was calculated by dividing the GAS counts obtained after H_2_O_2_ challenge with those obtained immediately prior challenge. Data represent the mean (±standard deviation) obtained from three independent determinations.

### Human blood phagocytosis assays

GAS sensitivity to phagocytic killing by human phagocytes was assessed using the Lancefield bactericidal test [19]. Approximately 10^4^ GAS cfu grown to early exponential phase (A_600nm = _0.15) in THY broth were rotated end-over-end at 37°C with heparinized human blood from healthy volunteers. Proliferation or killing of GAS was determined by comparing bacterial counts at 3 h post-incubation with those at time of inoculation (0 h), as obtained by quantitative culture of samples on tryptic soy-blood plates. Negative control assays consisted of GAS-blood suspensions incubated without rotation that is required for efficient phagocytosis. For positive control, an M-type 3 protein mutant [44] that shows reduced survival in human blood was assayed in parallel.

### Macrophage infection assays

Mouse RAW264.7 macrophages [45] were maintained in Dulbecco's modified Eagle's medium (DMEM, Gibco) supplemented with 10% fetal calf serum. For GAS infections, wild-type and perR mutant bacteria were grown to early exponential phase (A_600nm_ = 0.15–0.2), washed and suspended in DMEM (no serum), then used to infect macrophages grown in 24-well culture plates at 37°C in 5% CO_2_ at a multiplicity of infection of 0.5–1. Following 1 h infection, non-associated bacteria were removed by washing twice with the infected media and the cells were lifted with 0.25 ml H_2_O and lysed with an equal volume of 0.1% Triton X-100 (0.05% final concentration). Macrophage-associated (adherent and internalized) GAS counts were determined by plating serial dilutions of macrophage lysates on tryptic soy-blood agar. For determination of intracellular GAS counts, gentamicin-penicillin exclusion assays were performed. In brief, macrophages were infected for 1 h as described above and extracellular bacteria were killed by the addition of gentamicin-penicillin (200 µg/ml–20 µg/ml, respectively) for an additional hour. Antibiotics were removed with three PBS washes and intracellular GAS counts were determined in macrophage lysates obtained with Triton X-100 as described above. For certain experiments, the general oxidative burst inhibitor diphenyleneiodonium chloride (DPI) (Sigma) was added during the 1 h antibiotic treatment period at a final concentration of 10 µM. Data are presented as GAS counts recovered from each well (∼10^6^ macrophages) and represent the mean cfu (±standard deviation) obtained from three independent determinations each performed in duplicate. Reduced ROS production in DPI-treated macrophages, versus untreated cells as control, was confirmed using the ROS indicator dye dichlorodihydrofluorescein diacetate (H_2_-DCF-DA) (Invitrogen) at 10 µM final concentration. DCF fluorescence due to reaction with ROS inside macrophages was measured with a Synergy 2 fluorometer (Bio-Tek) with excitation and emission filters of 485±20 and 528±20 nm, respectively. DPI did not affect macrophage viability as monitored by trypan blue staining of DPI-treated and untreated cells.

### Baboon pharyngeal colonization

Experiments in baboons were performed at the primate center of the University of Oklahoma Health Sciences Center (OUHSC) as described with minor modifications [21]. Prior to inoculation, throat cultures of all animals used were confirmed GAS-negative. Groups of five baboons (2–2.7 y), matched for age and sex, were co-infected with a 1∶1 mixture of stationary phase-grown wild-type GAS strain 003Sm and integrant strain 003Sm-Int- *perR*Δ, or with wild-type strain 003Sm and integrant strain 003Sm-Int-WT, as a control group. Strains 003Sm-Int-*perR*Δ and 003Sm-Int-WT were derived by chromosomal integration of plasmid pJL*perR*Δ either upstream or downstream of *perR* (*spyM3_0147*) resulting in *perR* mutant and wild-type phenotype, respectively. Lack of PerR expression in strain 003Sm-Int-*perR*Δ and wild-type levels of PerR in strain 003Sm-Int-WT was confirmed both by qRT-PCR and immunoblotting of protein lysates of the two strains using anti-PerR rabbit antiserum. No loss of Cm resistance due to pJL*perR*Δ spontaneous excision from the chromosome could be detected in either of two integrant strains following overnight broth culture (16–18 h) at 37°C in the absence of antibiotics, as determined by quantitative culture of duplicate samples on blood agar carrying Sm or Cm. Thus, chromosomal integration of pJL*perR*Δ in the two integrant strains was stable. Neither of the two integrant strains showed any competitive advantage or disadvantage in growth when each of the two strains was co-cultured with wild-type strain 003Sm in THY broth *in vitro*, as determined by quantitative culture of samples obtained before and after co-culture on Sm or Cm.

For inoculation, animals were anaesthetized and the posterior pharynx infected by pipeting 1 ml of bacterial suspension carrying 1.5×10^8^ cfu of wild-type strain and an equivalent number of one of two integrant strains. Throat swabs were collected from anaesthetized animals at 1, 3, 24, 48, and 72 h and at 7, 14, 21, 28, 35 and 42 d post-inoculation. The bacteria on each swab were suspended in 2 ml of THY-10% glycerol, the suspension was mixed vigorously and immediately frozen on dry ice and stored at −80°C. Samples of the inoculum were processed similarly. Freezing of GAS in THY-10% glycerol preserves viability for at least 3 mo. The frozen samples were thawed, serially diluted, and plated in duplicate on THY-blood agar containing either streptomycin to determine the total GAS cfu in each sample, or chloramphenicol to determine the proportion of GAS cfu representing plasmid integrant strains 003Sm-Int-WT or 003Sm-Int-*perR*Δ. Each of the two antibiotics inhibited growth of the baboon pharyngeal microflora. The competitive colonization index (CCI) for each animal at each time point represents the cfu count of wild-type bacteria divided by the cfu count of the plasmid integrant strain studied. When no colonies were recovered for one of the two strains studied in each sample, the animal was considered negative for that strain and zero counts are shown. However, a cfu count of nine, and not zero, was used for CCI determination in these samples. Since the throat swab culture detection limit was 10 cfu/animal for each strain, 9 cfu would be the highest possible undetectable count and, therefore, such approach represents a conservative analytic strategy. A CCI value was not recorded and is not shown for swab samples that yielded no colonies of either of the two co-infecting strains. An animal was considered to have cleared a strain at the time point beyond which subsequent throat cultures did not yield any colonies of that strain.

### Microarray procedures and data analyses

The GAS DNA microarray used is a modified version of that reported previously [39] and represents all ORFs of M-type 1 GAS strain SF370, in addition to 71 M-type 18 and 21 M-type 3 specific ORFs. cDNA synthesis and labeling, microarray hybridizations and data analyses were performed as described [39]. Transcriptome comparisons were performed between wild-type strain 003Sm and *perR* mutant strain 003Sm*perR*Δ grown to mid- (A_600nm_ = 0.25) or late-exponential (A_600nm_ = 0.6) phase. For each strain, total bacterial RNA was extracted from four independent cultures, pooled and used for cDNA synthesis and labeling. In each experiment, the wild-type and mutant RNA samples to be compared were converted to cDNA and fluorescently labeled with Cy3 or Cy5 in direct (Cy3-Cy5) and dye swap (Cy5-Cy3) labeling reactions to correct for dye-dependent variation of labeling efficiency, and co-hybridized on the arrays. Statistical significance of the difference in mean fluorescence intensity calculated for each gene in the two RNA samples compared was determined by an unpaired 2-tailed Student T-test. Genes whose relative expression (fold-change) exceeded 1.6-fold and had *P* values ≤0.01 were considered as differentially expressed. The False Discovery Rate, estimated by the procedure described by the National Institute of Aging array analysis tool, was ≤0.05 ([46]; http://Igsun.grc.nia.gov/ANOVA/). The primary microarray data have been submitted to ArrayExpress database of the European Bioinformatics Institute under accession number E-MEXP-1625.

### Computational genome sequence analysis

The M-type 3 strain MGAS315 genome sequence [18] was searched for PerR [TTANAATNATTNTAA] and AdcR [TTAAC(T/C)(A/G)GTTAA] conserved motif sequences using both ACMES (Advanced Content Matching Engine for Sequences) [47] provided by the University of Missouri-Columbia and the PredictRegulon web server [48].

### Electrophoretic mobility shift assays (EMSAs)

Binding of recombinant PerR to DNA fragments representing putative PerR-regulated promoters was performed as described with modifications [8]. Promoter DNA fragments (220–300 bp) were obtained by PCR using wild-type strain 003Sm chromosome as template and primers described in Table S2. Purified PerR was diluted in TDG buffer (50 mM Tris, pH 8.0; 0.1 mM DTT; 5% glycerol) and was mixed with promoter DNA for 15 min at room temperature in 10 µl reactions that contained the following: 45 mM Tris (pH 8.0), 1 mM DTT, 50 ng/µl bovine serum albumin, 6.5% glycerol. Promoter DNA was added at a final concentration of 50 nM. Reactions were resolved on 6% native polyacrylamide DNA retardation gels (Invitrogen) at 100 V for 100 min and gels were stained with ethidium bromide. Negative control assays were carried out with the 246 bp promoter of *guaB*, encoding inosine monophosphate dehydrogenase [49], which is not PerR-regulated.

## Supporting Information

Figure S1 Survival of wild-type GAS strain 003Sm and its isogenic *perR* mutant strain 003Sm*perR*Δ following H_2_O_2_ challenge. Bacteria were grown to early-exponential phase and then challenged with H_2_O_2_ for 1 hr at a final concentration of 10 mM. Culture samples were removed before (0 min) and after (60 min) H_2_O_2_ challenge and the colony forming units (cfu) for each strain were determined by quantitative culture on tryptic soy-blood agar plates.
(404 KB TIF)

Figure S2 Baboon throat GAS colonization in groups of 5 animals coinfected with either wild-type strain 003Sm and *perR* mutant 003Sm-Int-*perR*Δ (A) or 003Sm and PerR-expressing strain 003Sm-Int-WT. Animals in each group were inoculated with a suspension carrying equivalent numbers of each of the two strains and eleven consecutive throat swabs were collected over 42 days at the time points indicated. Counts of each of the two co-infecting strains in each animal were determined by quantitative culture of GAS recovered on throat swabs; cfu counts on blood agar-streptomycin determined total GAS cfu, whereas counts on blood agar-chloramphenicol determined the proportion of GAS representing integrant strain 003Sm-Int-*perR*D or 003Sm-Int-WT in each of the two animal groups.
(978 KB TIF)

Table S1 PerR-regulated genes in M-type 3 GAS strain 003Sm.
(48 KB DOC)

Table S2 Oligonucleotide primers used in this study.
(50 KB DOC)
