# Supplementary figures and images for: PerR Confers Phagocytic Killing Resistance and Allows Pharyngeal Colonization by Group A Streptococcus
Source: PLoS Pathog. 2008 Sep 5;4(9):e1000145. doi: 10.1371/journal.ppat.1000145 (PMC2518855; doi:10.1371/journal.ppat.1000145)

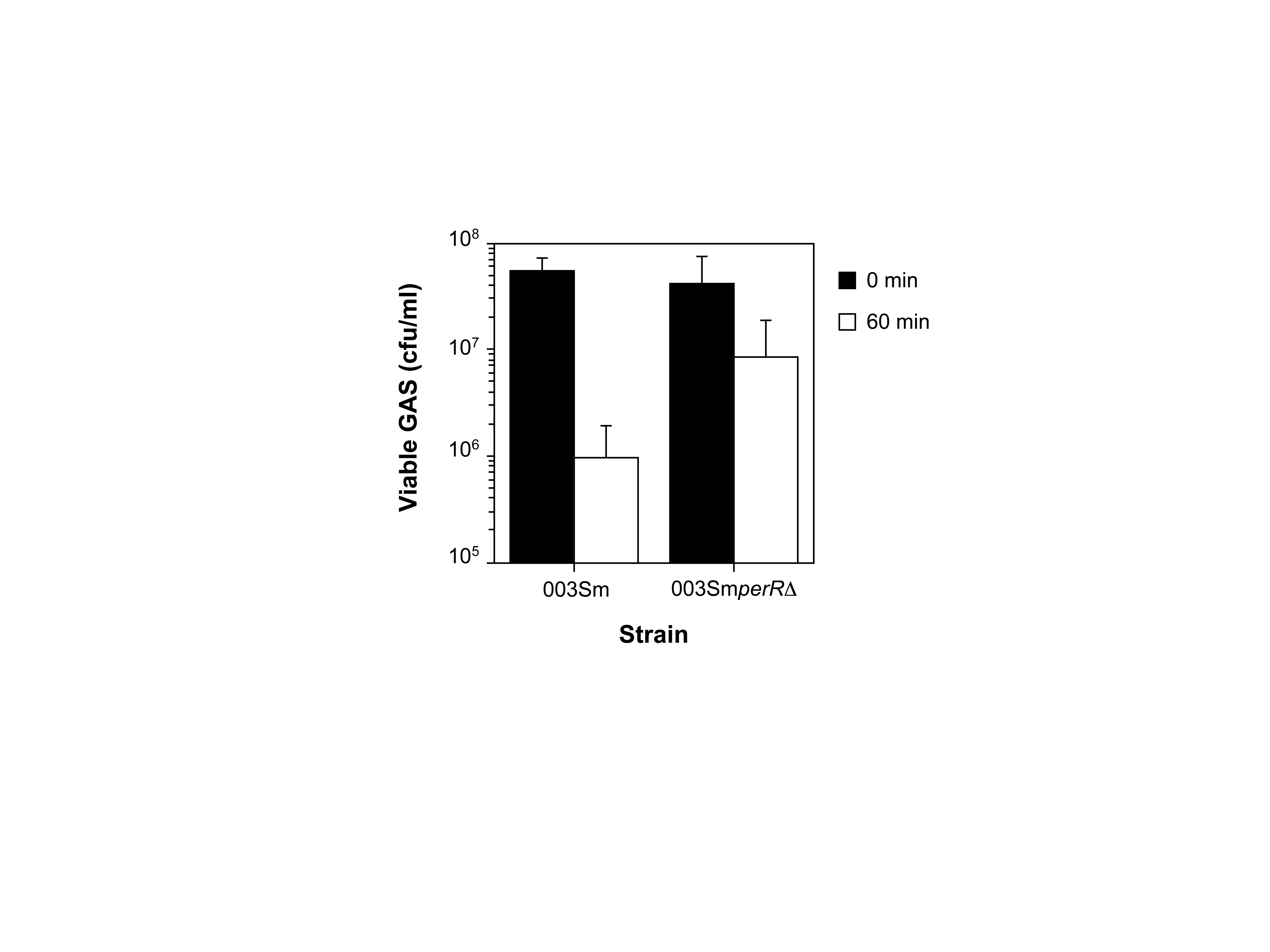

Supplement: Figure S1 — Survival of wild-type GAS strain 003Sm and its isogenic perR mutant strain 003SmperRΔ following H2O2 challenge. Bacteria were grown to early-exponential phase and then challenged with H2O2 for 1 hr at a final concentration of 10 mM. Culture samples were removed before (0 min) and after (60 min) H2O2 challenge and the colony forming units (cfu) for each strain were determined by quantitative culture on tryptic soy-blood agar plates. (404 KB TIF) [file ppat.1000145.s001.tif]

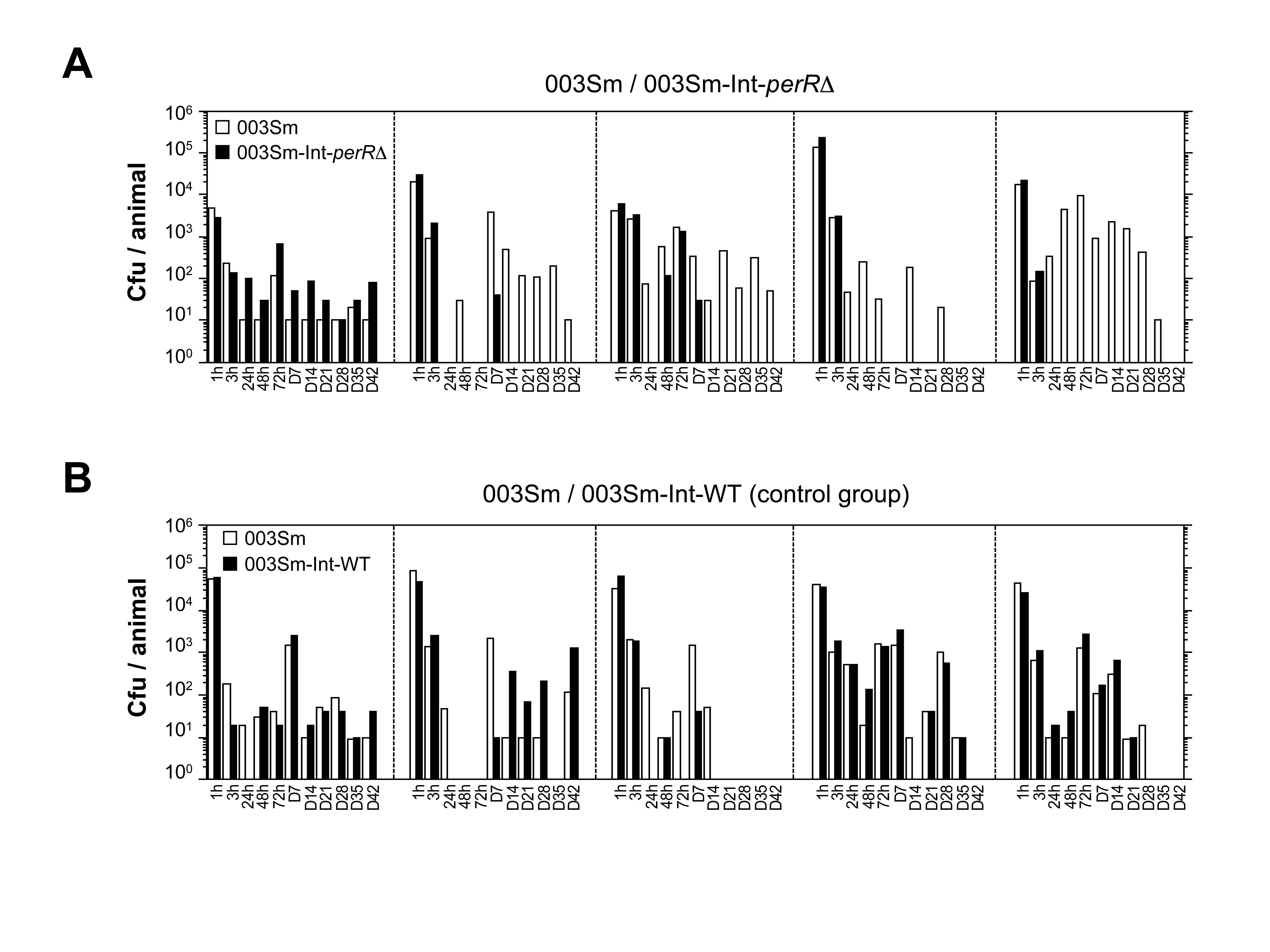

Supplement: Figure S2 — Baboon throat GAS colonization in groups of 5 animals coinfected with either wild-type strain 003Sm and perR mutant 003Sm-Int-perRΔ (A) or 003Sm and PerR-expressing strain 003Sm-Int-WT. Animals in each group were inoculated with a suspension carrying equivalent numbers of each of the two strains and eleven consecutive throat swabs were collected over 42 days at the time points indicated. Counts of each of the two co-infecting strains in each animal were determined by quantitative culture of GAS recovered on throat swabs; cfu counts on blood agar-streptomycin determined total GAS cfu, whereas counts on blood agar-chloramphenicol determined the proportion of GAS representing integrant strain 003Sm-Int-perRD or 003Sm-Int-WT in each of the two animal groups. (978 KB TIF) [file ppat.1000145.s002.tif]
