# Supplementary material for: PerR Confers Phagocytic Killing Resistance and Allows Pharyngeal Colonization by Group A Streptococcus
Source: PLoS Pathog. 2008 Sep 5;4(9):e1000145. doi: 10.1371/journal.ppat.1000145 (PMC2518855; doi:10.1371/journal.ppat.1000145)
Supplement: Table S1 — PerR-regulated genes in M-type 3 GAS strain 003Sm. (48 KB DOC) [file ppat.1000145.s003.doc]

**Supporting Information: Table S1.** PerR-regulated genes in M-type 3 GAS strain 003Sm.

|  | | | **Relative expression in wild type^c^** | |
| --- | --- | --- | --- | --- |
| **SPy no.^a^** | **Gene** | **Function^b^** | **Mid-exponential** | **Late-exponential** |
| ***Sugar metabolism/transport (17)*** | | | | |
| SPy0572 (0404) | *pts* | Beta-glucoside permease, PTS IIABC | -1.62 | +1.85 |
| SPy1058 (0742) | *pts* | Putative PTS enzyme IIB | -1.11 | +1.64 |
| SPy1060 (0744) | *pts* | Putative PTS enzyme IID | -1.48 | +1.67 |
| SPy1296 (0985) | *malG* | Putative ABC transport (permease) | -1.07 | +1.85 |
| SPy1707 (1484) | *lacB.1* | Galactose 6-phosphate isomerase | -2.35 | +1.43 |
| SPy1708 (1485) | *lacA.1* | Galactose 6-phosphate isomerase | -1.90 | +1.28 |
| SPy1710 (1487) | *pts* | Putative PTS enzyme IIB, galactose-specific | -1.46 | +2.94 |
| SPy1711 (1488) | *pts* | Putative PTS enzyme IIA, galactose-specific | -1.36 | +3.12 |
| SPy1712 (1489) | *lacR.1* | Putative lactose PTS repressor | -1.52 | +1.92 |
| SPy1916 (1653) | *lacG* | Putative phospho-beta-D- galactosidase | -1.01 | +1.67 |
| SPy1917 (1654) | *lacE* | Putative PTS, enzyme IIBC, lactose-specific | -1.69 | +4.0 |
| SPy1919 (1656) | *lacD.2* | Tagatose 1,6-diphosphate aldolase | -1.77 | +4.0 |
| SPy1921 (1657) | *lacC.2* | Putative tagatose 6-phosphate kinase | -1.65 | +5.26 |
| SPy1922 (1658) | *lacB.2* | Galactose 6-phosphate isomerase | -1.14 | +5.0 |
| SPy1923 (1659) | *lacA.2* | Galactose 6-phosphate isomerase | -1.21 | +4.76 |
| SPy1924 (1660) | *lacR.2* | Putative lactose PTS repressor | +1.18 | +1.89 |
| SPy1972 (1694) | *pulA* | Putative pullulanase (starch degradation) | +1.22 | +1.92 |
| ***General biosynthesis/metabolism (10)*** | | | | |
| SPy0033 (0027) | *purE* | Phosphoribosylaminoimidazole-carboxylase catalytic subunit | +2.22 | -1.04 |
| SPy0425 (0301) | *nrdF.2* | Ribonucleotide reductase (beta-subunit) | +1.14 | -2.21 |
| SPy0426 (0302) | *nrdl.2* | Putative ribonucleotide reductase | -1.11 | -2.66 |
| SPy0427 (0303) | *nrdE.2* | Ribonucleotide reductase (alpha-subunit) | -1.33 | -2.75 |
| SPy1183 (0830) |  | Acetyl-CoA carboxylase | +1.11 | +1.72 |
| SPy1542 (1192) | *arcT* | Xaa-His dipeptidase | +1.26 | +1.72 |
| SPy1543 (1193) | *arcD* | Arginine/ornithine antiporter | -1.87 | +1.49 |
| SPy1871 (1615) | *rpsN.2* | 30S subunit ribosomal protein S14 | -3.33 | -3.07 ^e^ |
| SPy2000 (1718) | *dppA* | Dipeptide ABC transport (lipoprotein) | -1.61 | -1.98 ^e^ |
| SPy2004 (1722) | *dppE* | Putative dipeptide ABC transporter | -1.27 | -1.64 |
| ***Oxidative stress - Virulence (putative) (6)*** | | | | |
| SPy0714 (0466) | *adcA* | Putative adhesin (Zinc-binding lipoprotein) | -4.62 | -3.74 ^e^ |
| SPy1159 (0815) | *hlyIII* | Putative hemolysin III | +3.03 | -1.21 |
| SPy1434 (1093) | *pmtA* | Putative heavy metal transport ATPase | -11.28 | -7.17 ^e^ |
| SPy2079 (1770) | *ahpC* | Alkyl hydroperoxidase | -1.54 | -1.72 |
| SPy2080 (1771) | *ahpF* | Alkyl hydroperoxidase reductase | -1.38 | -1.99 |
| n/a^d^ (1095) | *mf4* | Putative mitogenic factor - DNase (phage) | -1.89 | -2.16 ^e^ |
| ***Hypothetical (5)*** | | | | |
| SPy0116 (n/a^d^) |  | Hypothetical protein | -1.25 | -1.72 |
| SPy0710 (1208) |  | Conserved hypothetical protein (phage) | +2.0 | +1.04 |
| SPy1160 (0816) |  | Hypothetical protein | +3.85 | -1.31 |
| SPy2006 (1724) | *phtD* | Hypothetical protein | -1.27 | -2.30 |
| SPy2191 (1843) |  | Hypothetical protein | -1.01 | -1.97 |
| ***Metal - ABC transport (3)*** | | | | |
| SPy0092 (0069) | *adcR* | Putative repressor protein | -1.92 | -1.33 |
| SPy0093 (0070) | *adcC* | ABC transporter (ATP-binding protein) | -1.83 | -1.49 |
| SPy0094 (0071) | *adcB* | Putative ABC transporter (permease) | -1.73 | -1.53 |
| ***Miscellaneous (1)*** | | | | |
| SPy1856 (1601) | *norA* | Putative antibiotic resistance protein | -1.68 | -1.14 |

a. SPy ORF numbers of M-type 1 strain SF370 [32]; spyM3 ORF numbers of M-type 3 strain MGAS315 [18] in parentheses

b. NCBI annotation

c. Expression levels in wild type GAS compared to perR mutant 003Sm*perR*Δ

d. Non-applicable, *spyM3_1095* only present in M-type 3 GAS.

e. Similar regulation pattern in both mid- and late-exponential phase
