# Supplementary material for: PerR Confers Phagocytic Killing Resistance and Allows Pharyngeal Colonization by Group A Streptococcus
Source: PLoS Pathog. 2008 Sep 5;4(9):e1000145. doi: 10.1371/journal.ppat.1000145 (PMC2518855; doi:10.1371/journal.ppat.1000145)
Supplement: Table S2 — Oligonucleotide primers used in this study. (50 KB DOC) [file ppat.1000145.s004.doc]

**Supporting Information: Table S2.** Oligonucleotide primers used in this study.

| **Primer^a^** | **Sequence (5’-3’)^a^** | **Position^b^** | **Reference** |
| --- | --- | --- | --- |
| ***PCR and RT-PCR*** | | | |
| perR-F(overlap)^c^ | atggacattcattcacatcagcaa  *gaccaacctgatttttagagttaa* | 1-24;  *451-468* | This study |
| perR-R | ttgctgatgtgaatgaatgtccat | 24-1 | This study |
| perR-F (*Bam*HI) | cgcggatccgacattcattcacatcagcaagcc | 4-27 | This study |
| perR-R (*Hin*dIII) | cccaagcttttaactctaaaaatcaggttggtcttttgc | 468-445 | This study |
| perR-F-RBS (*Bam*HI) | ccgggatccaaaaaaggataggagtagcagctt | (-24)-(-1) | This study |
| perR-R (*Pst*I) | gcactgcagttaactctaaaaatcaggttggtc | 468-450 | This study |
| 0146-F (*Bam*HI) | cgcggatccaaatagataggggaacaagtgatt | (-18)-6 | This study |
| 0148-R (*Eco*RV) | ccggatatccctttccggactttcagcgtt | 410-390 | This study |
| rpsL-F | atgtagatgcctacaattaaccaattg | (-6)-21 | 43 |
| rpsL-R | tacgactcatttctctttatcccct | 443-419 | 43 |
| ***qRT-PCR*** | | | |
| rt0027-F (*purE*) | tcaaaatcatcatcgcagga | 296-315 | This study |
| rt0027-R | acgtgatttgacaggcacac | 396-377 | This study |
| rt0069-F (*adcR*) | tggagcttgtcaaagtgacg | 81-100 | This study |
| rt0069-R | ccttagttactgccgcctga | 211-192 | This study |
| rt0146-F | gcaattccctttgaggttga | 217-236 | This study |
| rt0146-R | ccaaaagactttggcctgac | 318-299 | This study |
| rt0147-F (*perR*) | taacatgagccttgccacag | 177-196 | This study |
| rt0147-R | ccacattgacgtgttgatgg | 307-288 | This study |
| rt0148-F | ttttgccgaatcaacaacag | 267-286 | This study |
| rt0148-R | tgaaagtccggaaaggaaaa | 405-386 | This study |
| rt0302-F (*nrdI.2*) | ataacacccatcgcttcgtc | 41-60 | This study |
| rt0302-R | cagctgcataggtaggcaca | 157-138 | This study |
| rt0466-F (*adcA*) | agcagagcatttccacctgta | 1416-1435 | This study |
| rt0466-R | atttcttgggcaatttcacg | 1535-1516 | This study |
| rt0815-F (*hylIII*) | actccatggcttatggttcg | 212-231 | This study |
| rt0815-R | acaagggataaggcaacagg | 320-301 | This study |
| rt1093-F (*pmtA*) | ttcgtaaaggcgaagctgttc | 446-466 | This study |
| rt1093-R | atcctggccttctgccttat | 561-542 | This study |
| rt1095-F (*mf4*) | ctgaagctttgtcgtggaca | 155-174 | This study |
| rt1095-R | ctatttggccttttggtgga | 256-237 | This study |
| rt1208-F | aggggatacagtcacgcaac | 897-916 | This study |
| rt1208-R | acctacaacctcgctggcta | 1041-1022 | This study |
| rt1484-F (*lacB*.1) | gtggaactggagttggcatta | 194-214 | This study |
| rt1484-R | ccaatcacattggcattcaa | 314-195 | This study |
| rt1487-F | tcgtggctatgatgtgcattg | 84-104 | This study |
| rt1487-R | actgtgggaattggcatctc | 212-193 | This study |
| rt1489-F (*lacR*.1) | tgaccaatagcctccctgtc | 359-378 | This study |
| rt1489-R | gggaccctacaaaagctcctg | 466-446 | This study |
| rt1615-F (*rpsN*.2) | agtacgctgaccttcgtcgtg | 56-76 | This study |
| rt1615-R | cgtcaattttgtcgcggttc | 166-147 | This study |
| rt1654-F (*lacE*) | agccattgcagcgattactta | 747-767 | This study |
| rt1654-R | cgacgagagttgcaccagta | 886-867 | This study |
| rt1658-F (*lacB*.2) | cctggaattcgttcagcactc | 235-255 | This study |
| rt1658-R | accgatgatttttccaccaa | 336-317 | This study |
| rt1660-F (*lacR*.2) | attggtggtgaatgtcgtga | 433-414 | This study |
| rt1660-R | cagcatttgcgctaacaaaa | 538-519 | This study |
| rt1724-F (*phtD*) | tattgagccacgacttgctg | 1659-1678 | This study |
| rt1724-R | tgaatacggaacgacatgga | 1782-1763 | This study |
| rt1770-F (*ahpC*) | tgacgctagcaccttgattg | 426-445 | This study |
| rt1770-R | gtcaaagtttcagcgccttc | 530-511 | This study |
| rt1800-F (*recA*) | tgattctggtgcggttgatc | 282-301 | 39 |
| rt1800-R | atttacgcatggcctgactc | 415-396 | 39 |
| rt1852-F (*hasB*) | tccccaaacgctaattgaag | 825-844 | 39 |
| rt1852-R | ttaaacggtaaaccccgact | 952-933 | 39 |
| ***Electrophoretic Mobility Shift Assays*** | | | |
| GS-1093-F (*pmtA*) | gccaagttctaaagcatgcac | (-181)-(-162) | This study |
| GS-1093-R | ccaaacacgctaaagtctcca | 61-41 | This study |
| GS-1488-F | catctaaggggctcctttct | (-239)-(-220) | This study |
| GS-1488-R | tgcgtccttgcttcagtaaa | 38-19 | This study |
| GS-1659-F (*lacA*.2) | tggtgggtgtatgattaggtca | (-125)-(-105) | This study |
| GS-1659-R | acttcgttttcactaacatca | 110-90 | This study |
| GS-1660-F (*lacR*.2) | aatccctttgacagccactc | (-204)-(-185) | This study |
| GS-1660-R | gcggtcatatctgagatgttca | 107-86 | This study |
| GS-1770-F (*ahpC*) | gttgccttaacggaatcaaa | (-263)-(-244) | This study |
| GS-1770-R2 | caacaagattatgtctactcagaaacc | (-48)-(-74) | This study |
| GS-guaB-F | ctttacaggatagtagagattatgttt | (-233)-(-207) | This study |
| GS-guaB-R (*Nhe*I) | tttagctagccatccagataatcctcttttctt | 3-(-20) | This study |

a. Restriction sites in parentheses underlined; numbers for primers used in qRT-PCR and EMSAs indicate M-type 3 strain

MGAS315 ORFs [18]; F = Forward; R = Reverse

b. Nucleotide position relative to the start codon

c. Composite primer of 5' and 3'-end sequence of *perR* used in *perR* mutagenesis; 3'-end sequence italicized
